# Propofol postpones colorectal cancer development through circ_0026344/miR-645/Akt/mTOR signal pathway

**DOI:** 10.1515/med-2021-0254

**Published:** 2021-04-07

**Authors:** Xiaomin Cui, Jiying Feng, Jian Wu, Xiaobao Zhang, Mengyao Ding

**Affiliations:** Department of Postanesthesia Care Unit, The Affiliated Hospital of Kangda College of Nanjing Medical University (The First People’s Hospital of Lianyungang), Lianyungang, Jiangsu, China; Department of Anesthesiology, The Affiliated Hospital of Kangda College of Nanjing Medical University (The First People’s Hospital of Lianyungang), Lianyungang, Jiangsu, China; Department of Emergency, The First People’s Hospital of Lianyungang, Lianyungang, Jiangsu, China; Department of Anesthesiology, The Affiliated Hospital of Kangda College of Nanjing Medical University (The First People’s Hospital of Lianyungang), No. 188 Jianshe East Road, Lianyungang, 222002, Jiangsu, China

**Keywords:** colorectal cancer, circ_0026344, miR-645, Akt/mTOR, propofol

## Abstract

Colorectal cancer (CRC) is responsible for thousands of slow and painful annual deaths. Propofol, an anesthetic, is commonly used in CRC surgery. The role of circularRNA0026344 (circ_0026344) in propofol-treated CRC remains unclear, which was further explored in this study. Real-time polymerase chain reaction (qPCR) was used to detect the expression of circ_0026344 and microRNA645 (miR-645) in CRC cells and normal cells. Western blot was devoted to testing the protein expression of phospho-protein kinase B (p-AKT), AKT, phospho-mammalian target of rapamycin (p-mTOR), and mTOR in CRC cells. Moreover, cell counting kit-8 (CCK8), colony formation, flow cytometry, and transwell assays were employed to assess the proliferation, apoptosis, and metastasis in CRC cells. Circinteractome online tool was applied to predict the combination between circ_0026344 and miR-645, which was further verified by dual-luciferase reporter system. circ_0026344 was lowly expressed and miR-645 was abundantly expressed in CRC cells. The relative protein expression of p-AKT/AKT and p-mTOR/mTOR was strikingly elevated by si-circ#1, which could be reversed by anti-miR-645 in propofol-treated CRC cells. circ_0026344 overexpression inhibited the proliferation and metastasis and promoted apoptosis in CRC cells. Propofol treatment induced the restraint in proliferation and metastasis and stimulation in apoptosis, which were allayed by si-circ#1; meanwhile, this alleviation could further be abolished by anti-miR-645 in CRC cells. Furthermore, circ_0026344 sponged miR-645 to inhibited Akt/mTOR signal pathway in propofol-treated CRC cells. Propofol postponed CRC process by circ_0026344/miR-645/Akt/mTOR axis. This finding might provide a possibility to improve the therapy of CRC with propofol.

## 1 Introduction

Colorectal cancer (CRC) deserved a seat among malignant tumors due to the third-ranking of incidence (10.2%) and the second-ranking mortality (9.2%) over the world in 2018 [1]. Studies looked at the incidence characters of CRC and exposed that CRC possessed the sporadic, inherited, and familial features [2,3,4]. For the sporadic CRC, lifestyle and living environment assumed the main responsibility; however, genetic factors have mastered the inherited and familial CRC [5]. CRC therapy has centered on the surgery due to the unsatisfying diagnosis that 80% CRC patients were judged as local stage [6]. Surgery tends to bring anguish to CRC patients, which is readily solved by the application of anesthetic. As an agent, 2,6-diisopropylphenyl (propofol) is extensively used in clinic to induct and maintain anesthesia [7]. The efficacy of propofol was not only reflected in the anesthesia, but also embodied in the affection of cancer process [8]. The inhibition of HIF-1α pathway was realized by the application of propofol in prostate cancer [9]. Propofol treatment induced the promotion of the proliferation and inhibition in the apoptosis in natural killer cells in colon cancer [10]. In bladder cancer cells, propofol triggered the facilitation in proliferation, metastasis, and the inhibition in apoptosis [11]. Propofol put off the progression of CRC by regulating STAT3/HOTAIR/WIF-1/Wnt axis [12]. These documents conveyed the information about the functional role of propofol in cancers; however, the regulatory mechanism of propofol in CRC is still fuzzy.

As single-stranded covalently closed RNAs, circular RNAs (circRNAs) have been recognized as participators in various diseases [13,14,15]. Has_circ_0000520 was lowly expressed in gastric cancer cells, and this downregulation was negatively relevant to tumor node metastasis (TNM) stage [16]. The dysregulation of circ-LDLRAD3 was related to the metastasis in pancreatic cancer cells [17]. The interaction between circRNAs and micro RNA (miRNAs)/message RNAs (mRNAs) attracted great attention of researchers in exploring the regulatory mechanism of circRNAs in cancers. circ-TTBK2 boosted glioma development by regulating miR-761/ITGB8 pathway [18]. circ-SERPINE2 facilitated the proliferation and repressed the apoptosis by miR-375/YWHAZ axis in gastric cancer [19]. For the underlying way of circRNA in propofol-treated CRC, the available information was limited, which was further studied in this project.

During the development of CRC, signal pathways play a crucial role. Protein kinase B (AKT)/mammalian target of rapamycin (mTOR) signal pathway was taking charge of CRC biology including proliferation, apoptosis, and metastasis [20,21]. As the upstream stimulating factor of mTOR, AKT can be activated into the phosphorylated state, which further induces the activation of mTOR [22]. The activation of AKT/mTOR led to repress the autophagy in myocardial injury [23]. GHET1 facilitated gastric cancer process by activating AKT/mTOR pathway [24]. ATL-1-induced suppression in AKT/mTOR pathway was associated with the postponement of CRC process [25]. However, AKT/mTOR pathway that participated in propofol-treated CRC progress continues to be unknown.

In the present study, the downregulation of circ_0026344 was founded in CRC cells. circ_0026344 overexpression suppressed the proliferation and metastasis and boosted the apoptosis in CRC cells. Meanwhile, circ_0026344 knockdown allayed propofol treatment-induced inhibition in proliferation, metastasis, and promotion of apoptosis in CRC cells. Moreover, circ_0026344 sponged miR-645 to regulate AKT/mTOR signal pathway in propofol-treated CRC cells. The circ_0026344/miR-645/AKT/mTOR pathway might conduce to improve CRC treatment.

## 2 Materials and methods

### 2.1 Cells and treatment

Normal human colonic epithelial cells (NCM460) were purchased from INCELL Corporation LLC (San Antonio, Texas, USA). Colon adenocarcinoma cells (SW480 and LOVO) were obtained from American Type Culture Collection (Rockville, MD, USA). Cells were seeded into Dulbecco’s Modified Eagle’s Medium (DMEM, Gibco, Carlsbad, CA, USA), containing 10% fetal bovine serum (FBS, Gibco) and 100 U/mL penicillin (Sigma-Aldrich, St. Louis, MO, USA) and 100 µg/mL streptomycin (Sigma-Aldrich) with 5% CO_2_ at 37°C. For the assessment of the effect of propofol on CRC cells, 0, 5, and 10 μg/mL of propofol (Sigma-Aldrich) were applied to treat CRC cells.

### 2.2 Cell transfection

SW480 and LOVO cells were cultured in 6-well plates for 12 h. After that, the transient transfection was performed by Lipofectomine 3000 transfection reagent (Invitrogen, Carlsbad, CA, USA) in SW480 and LOVO cells. Small interfering RNA targeting circ_0026344 (si-circ_0026344 (si-circ#1, si-circ#2, si-circ#3)), circ_0026344 overexpression (circ_0026344), miR-645 overexpression (miR-645), miR-645 inhibition (anti-miR-645), and its negative controls (si-NC, vector, miR-NC, anti-NC) were synthesized and provided by Ribobio (Guangzhou, China).

### 2.3 Cell counting Kit-8 (CCK8) assay

SW480 and LOVO cells (100 μL) were cultured in 96-well plates at 37°C for 12 h. CCK8 assay (Beyotime, Shanghai, China) was applied to evaluate the viability of SW480 and LOVO cells following the protocols. Following that, 100 μL of CCK8 was added into each well. Spectra Max 250 spectrophotometer (Molecular Devices, Sunnyvale, CA, USA) was applied to detect the absorbance at 450 nm at the predetermined time of 0, 24, 48, and 72 h after the incubation.

### 2.4 Colony formation assay

SW480 and LOVO cells were seeded into 6-well plates until the appearance of visible clones at 37°C with 5% CO_2_. Following that, cells were washed by phosphate buffer saline (PBS, Sigma-Aldrich), then blocked by methanol (Sigma-Aldrich) and stained by 0.05% crystal violet (Sigma-Aldrich) for 30 min each. The colonies were visualized by a camera (Nikon, Tokyo, Japan).

### 2.5 Flow cytometry assay

SW480 and LOVO cells were collected and suspended in cold PBS (Sigma-Aldrich). Following that, Annexin V-Fluorescein isothiocyanate (FITC)/Propidium lodide (PI) Apoptosis Kit (BD Biosciences, San Jose, CA, USA) was used to measure the apoptosis upon the guidance of the corresponding instruction. BD FACS Canto™ II flow cytometer (BD Biosciences) was employed to detect the apoptosis.

### 2.6 Transwell assay

The migration and invasion of SW480 and LOVO cells were assessed by transwell assay. For the detection of invasion, transwell chamber (8.0 μm pore, Corning, Corning, NY, USA) with the application of Matrigel coating (BD Biosciences) was devoted. For the migration assay, transwell chambers (Corning) without Matrigel coating (BD Biosciences) were applied. Cells were seeded into the upper chamber and the DMEM (Gibco) medium and 10% FBS (Gibco) was added into the lower chamber. Whether the migration assay or invasion assay, cells were cultured in an incubator with the setting of 37°C, 5% CO_2_, 24 h. Subsequently, cells were blocked and stained by 4% paraformaldehyde (Sigma-Aldrich) and 0.1% crystal violet (Sigma-Aldrich). Data were captured by an inverted microscope (magnification, ×100, CarlZeiss, Hallbergnoos, Germany).

### 2.7 RNA isolation and real-time polymerase chain reaction (qPCR)

TRIzol reagent (Thermo Fisher Scientific, Waltham, MA, USA) was applied to isolate the total RNA from SW480 and LOVO cells. Then, the RNA was reversed into cDNA by cDNA reverse transcription kit (Applied Biosystems, Foster City, CA, USA). Next, SYBR Green Master Mix kit (Takara, Dalian, China) and ABI7500 Real-time PCR system (Applied Biosystems) were employed to detect the relative expression of the interested RNAs, which was calculated by the method of 2^−ΔΔCt^. The primers used in this research were provided by Beijing Genomics Institute (BGI, Shenzhen, China) and listed as: circ_0026344, forward, 5′-CGTACCTGGAGACGCTGTTT-3′, reverse, 5′-GGGTTTGGGTACCAGCACT-3′; miR-645, forward, 5′-TCTAGGCTGGTACTGCT-3′, reverse, 5′-GAACATGTCTGCGTATCTC-3′; Glyceraldehyde 3-phosphate dehydrogenase (GAPDH), forward, 5′-CAGTCAGCCGCATCTTCTTTT-3′, reverse, 5′-GTGACCAGGCGCCCAATAC-3′; U6 small nuclear RNA (U6), forward, 5′-CTCGCTTCGGCAGCACA-3′, reverse, 5′-AACGCTTCACGAATTTGCGT-3′. GAPDH and U6 were recognized as the reference of circ_0026344 and miR-645, respectively.

### 2.8 Dual-luciferase reporter system

Dual-luciferase reporter system was devoted to confirming the prediction which was presented by Circinteractome online database about the combination between circ_0026344 and miR-645. The complementary sequence or the corresponding mutated sequence between circ_0026344 and miR-645 was cloned into pmirGLO vector (Promega, Madison, WI, USA) and named as circ_0026344 WT or circ_0026344 MUT. Then, the circ_0026344 WT or circ_0026344 MUT reporters were transfected into SW480 and LOVO cells with miR-645 or miR-NC. Dual-Luciferase Reporter Assay Kit (Promega) was used to determine the luciferase activity following the protocol.

### 2.9 Western blot

SW480 and LOVO cells were lysed by RIPA buffer (Beyotime). Following that, proteins were loaded onto sodium dodecyl sulfate polyacrylamidegel electrophoresis (SDS-PAGE, Beyotime) and polyvinylidene difluoride membrane (PVDF, Millipore, Boston, MA, USA). After washed by PBS (Sigma-Aldrich) for three times and blocked by nonfat milk, the membranes were incubated with the primary antibodies: phospho-protein kinase B (p-AKT, 1:2,000, Sigma-Aldrich) with the phosphorylation site in p308, AKT (1:1,000, Sigma-Aldrich), phospho-mammalian target of rapamycin (p-mTOR, 1:1,000, Sigma-Aldrich) with the phosphorylation site in S2481 and mTOR (1:1,000, Cell Signaling Technologies, Danvers, MA, USA) and GAPDH (1:1,000, Cell Signaling Technologies), and the second antibody: anti-rabbit IgG (1:5,000, Ruiyingbio, Shanghai, China), sequentially. ECL Detection Reagents (Amersham Biosciences, Piscataway, NJ, USA) were applied to determine the result. GAPDH served as a reference.

### 2.10 Data analysis

GraphPad software 6.0 (GraphPad Inc., San Diego, CA, USA) was adopted to analyze data. Each group of data was obtained from triplicate experiments and performed as mean ± standard deviation (SD). Between-group variance was presented by Student’s *t*-test or one-way Analysis of Variance (ANOVA). *P* < 0.05 was recognized as statistical significance.

## 3 Results

### 3.1 Propofol impaired the proliferation and metastasis and promoted the apoptosis in CRC cells

**Figure 1:**
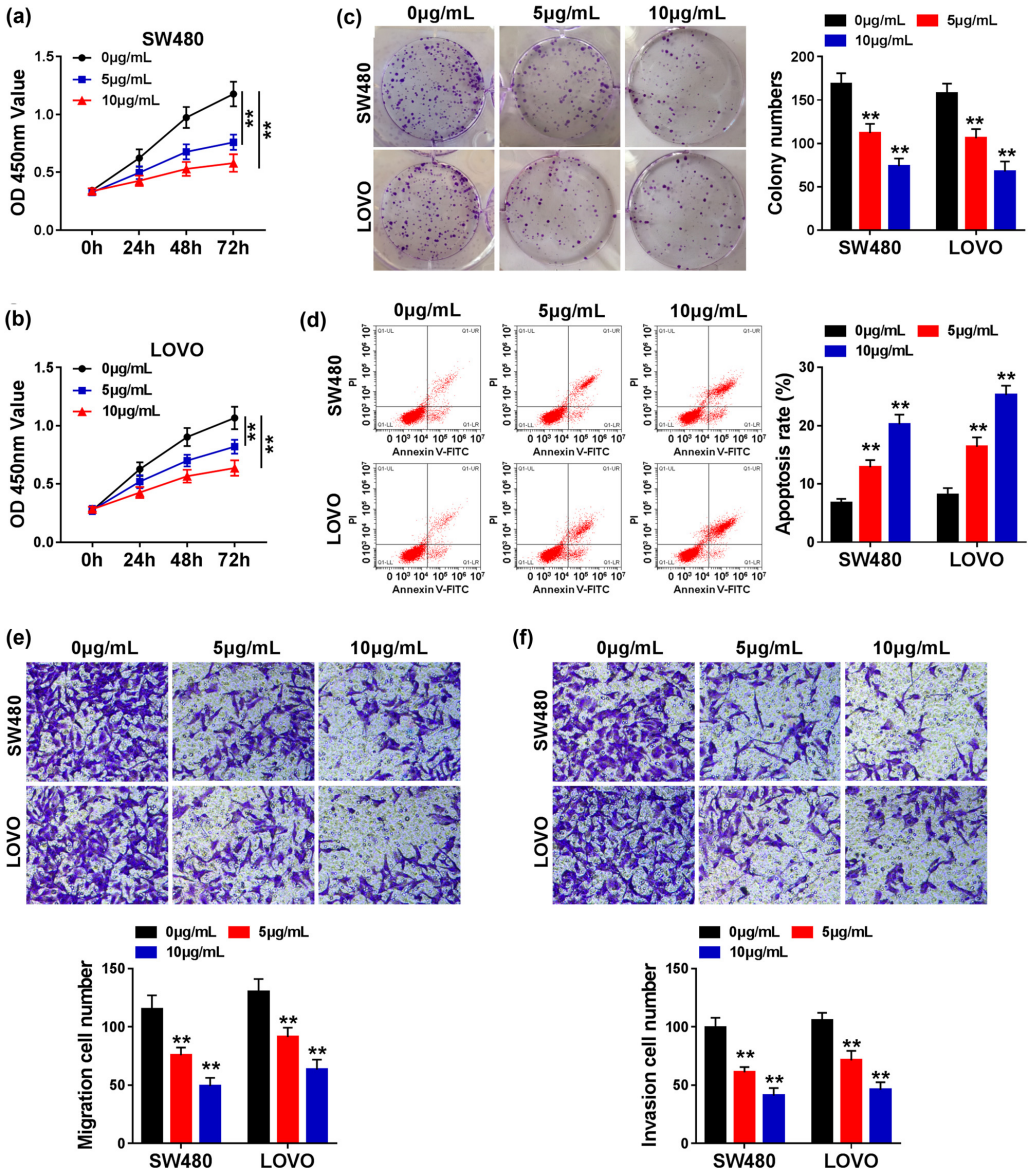
Propofol repressed cell proliferation, migration, and invasion, whereas induced cell apoptosis in SW480 and LOVO cells. SW480 and LOVO cells were treated with various doses of propofol (0, 5, and 10 μg/mL). (a–c) Cell proliferation was determined by CCK8 and colony formation assays. (d) Cell apoptosis was demonstrated by flow cytometry assay. (e and f) Cell migration and invasion were detected by transwell assay. Significant differences were compared with ANOVA. **P < 0.01.

Due to the frequent discovery in the association between propofol and CRC, the direct evidence in the effect of propofol on CRC was carried out in this study. The viability of SW480 and LOVO cells showed a time-dependent increase and a dose-dependent decrease in the treatment of propofol (Figure 1a and b). For the clonality, propofol also led to a dose-dependent inhibition in SW480 and LOVO cells (Figure 1c). A significant promotion of apoptosis was associated with the higher concentration propofol treatment in CRC cells (Figure 1d). As expected, the ability of migration and invasion presented a dose-dependent impairment in the treatment of propofol in CRC cells (Figure 1e and f). These data highlighted that propofol treatment restrained the proliferation and metastasis and triggered the apoptosis in CRC cells *in vitro*.

### 3.2 circ_0026344 overexpression inhibited the proliferation and metastasis and facilitated the apoptosis in CRC cells

**Figure 2:**
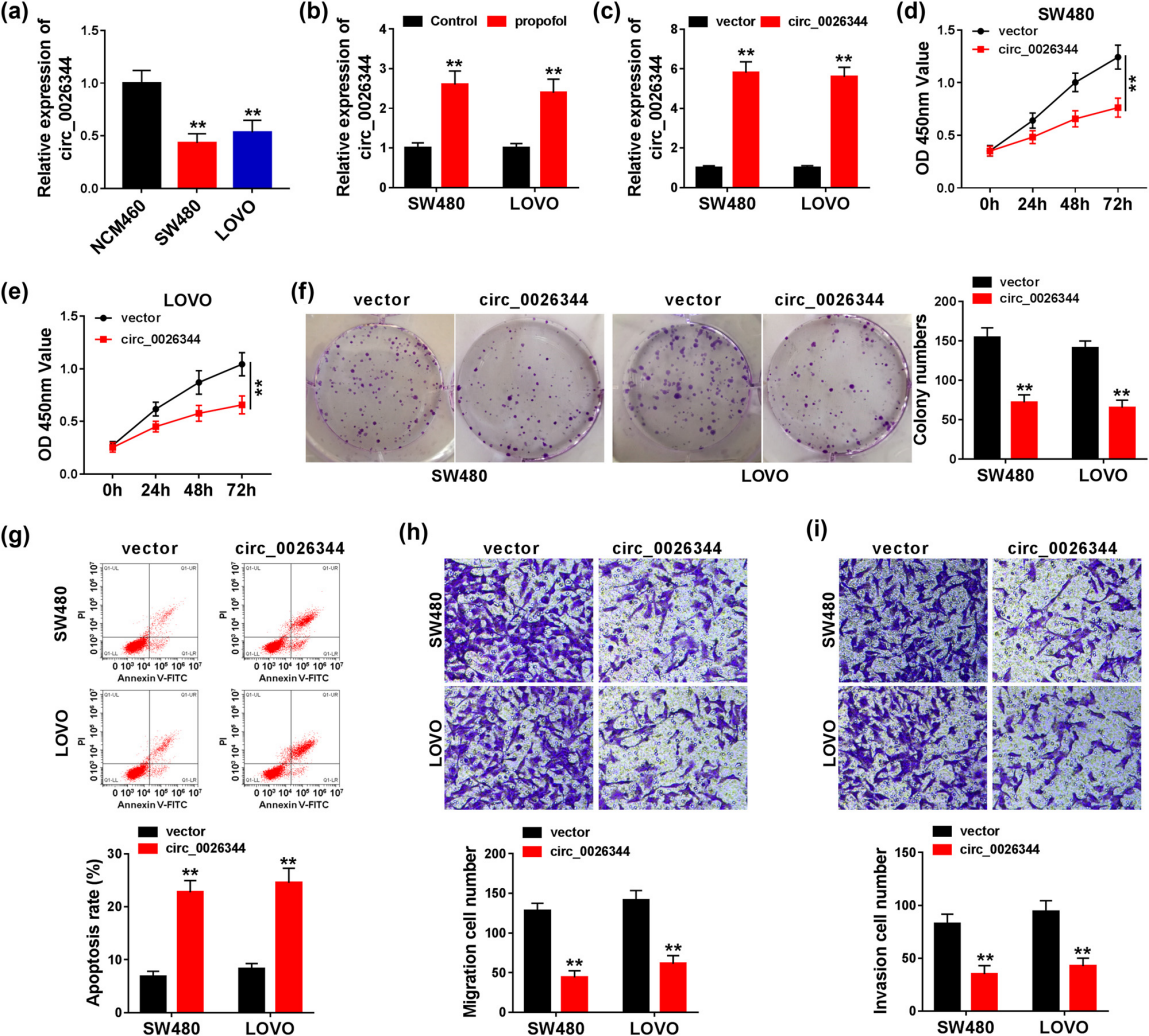
circ_0026344 overexpression inhibited CRC progression. (a) circ_0026344 expression was detected by qPCR in NCM460, SW480, and LOVO cells. (b) The effect of propofol on circ_0026344 expression was determined by qPCR in SW480 and LOVO cells. (c) The transfection efficiency of circ_0026344 was determined by qPCR in SW480 and LOVO cells. (d–f) The impact of circ_0026344 overexpression on cell proliferation was revealed by CCK8 and colony formation assays. (g) The impact of ectopic circ_0026344 expression on cell apoptosis was presented by flow cytometry assay. (h and i) The effects of circ_0026344 overexpression on the migration and invasion of SW480 and LOVO cells were disclosed by transwell assay. Significant differences were compared with Student’s *t*-test (b–i) or ANOVA (A). **P < 0.01.

Based on the role of propofol in CRC, we further explored the potential regulatory factors in propofol-treated CRC. circ_0026344 was conspicuously downregulated in CRC cells compared with normal cells (Figure 2a). Interestingly, the striking increase in circ_0026344 expression was induced by propofol in CRC cells (Figure 2b). This phenomenon inspired us to further study the relationship between circ_0026344 and propofol treatment in CRC. CRC cells were transfected with circ_0026344 or vector, and the successful transfection efficiency was exposed by the notable increase in circ_0026344 expression in CRC cells (Figure 2c). Moreover, circ_0026344 overexpression triggered the restraint in proliferation, metastasis, and the promotion of apoptosis (Figure 2d–i). These data exposed that circ_0026344 might serve as a suppressor in CRC.

### 3.3 circ_0026344 knockdown allayed propofol-induced restraint in proliferation, metastasis, and promotion of apoptosis in CRC cells

**Figure 3:**
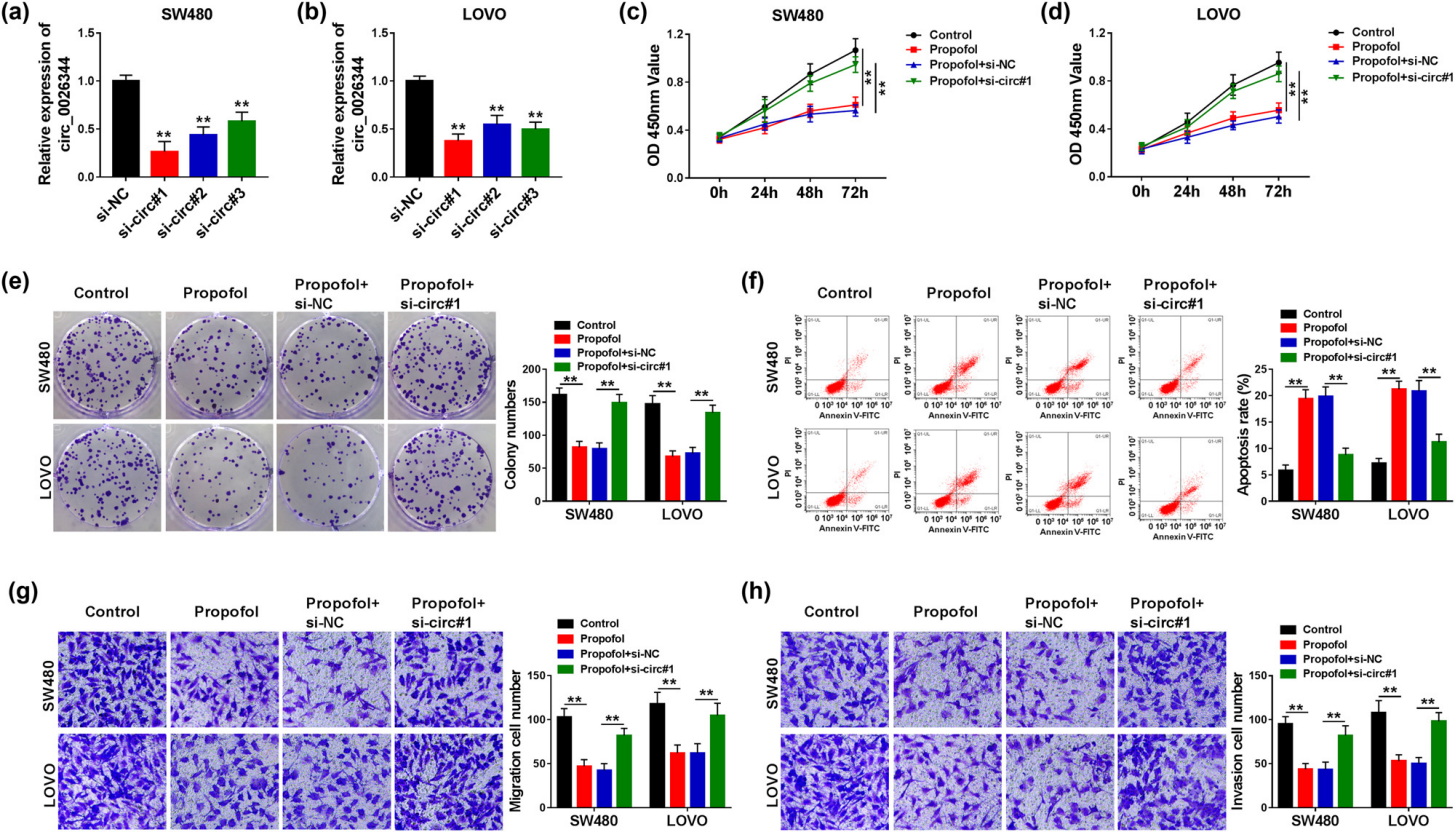
circ_0026344 silencing attenuated propofol-mediated effects on CRC progression. (a and b) The interfering efficiency of si-circ#1, si-circ#2, and si-circ#3 was determined by qPCR in SW480 and LOVO cells. (c–e) The impacts between propofol treatment and circ_0026344 silencing on cell proliferation were explained by CCK8 and colony formation assays. (f) The influences between propofol treatment and circ_0026344 knockdown on the apoptosis of SW480 and LOVO cells were presented by flow cytometry analysis. (g and h) The influences between propofol treatment and circ_0026344 absence on the migration and invasion of SW480 and LOVO cells were revealed by transwell assay. Significant differences were compared with ANOVA. **P < 0.01.

Given the potential role of circ_0026344 in CRC, the interaction between circ_0026344 and propofol treatment was further studied by loss-of-function approaches. The knockdown efficiency of circ_0026344 was exposed by the conspicuous decrease in circ_0026344 expression in CRC cells (Figure 3a and b). Due to the preeminent performance, si-circ#1 was employed to the following tests. In terms of the proliferation, propofol treatment led to a noticeable suppression, which was ameliorated by si-circ#1 in CRC cells (Figure 3c–e). However, si-circ#1 reversed propofol-mediated facilitation in apoptosis in CRC cells (Figure 3f). For the metastasis, si-circ#1 possessed the ability to attenuate propofol-induced restraint in the migration and invasion in CRC cells (Figure 3g and h). These results clarified that circ_0026344 deletion impaired the efficacy of propofol in CRC cells *in vitro*.

### 3.4 circ_0026344 sponged miR-645

**Figure 4:**
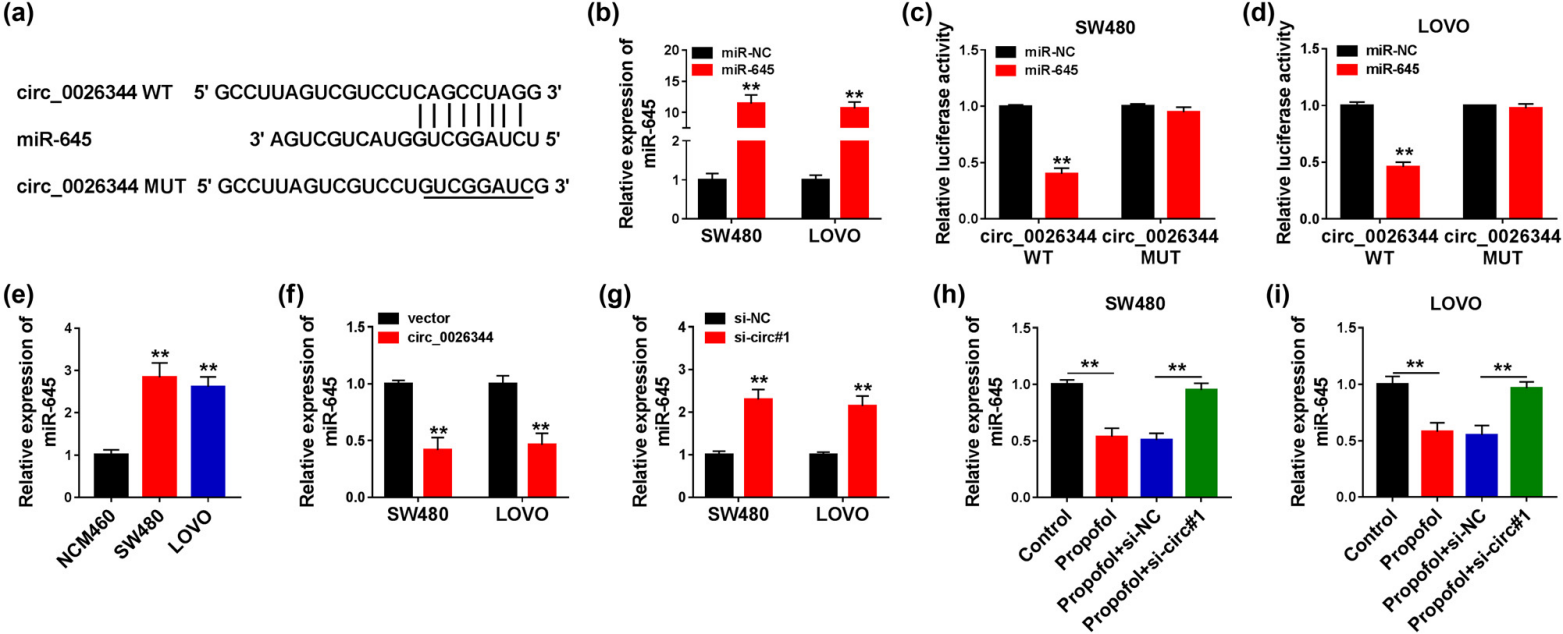
circ_0026344 was associated with miR-645 in SW480 and LOVO cells. (a) Circinteractome online database was employed to predict the putative binding sites of circ_0026344 in miR-645. (b) The transfection efficiency of miR-645 was determined by qPCR in SW480 and LOVO cells. (c and d) Luciferase activities were detected by dual-luciferase reporter assay in SW480 and LOVO cells. (e) miR-645 expression was determined by qPCR in NCM460, SW480, and LOVO cells. (f and g) The effects of circ_0026344 overexpression and silencing on miR-645 expression were revealed by qPCR in SW480 and LOVO cells. (h and i) The impacts between propofol treatment and circ_0026344 silencing on miR-645 expression were demonstrated by qPCR in SW480 and LOVO cells. Significant differences were compared with Student’s *t*-test (b, c, d, f, and g) or ANOVA (e, h, and i). **P < 0.01.

According to the above results, we wondered whether circ_0026344 participated in propofol-treated CRC progression by regulating the downstream factors. Thus, the underlying miRNA sponged by circ_0026344 was further studied. The combination sequence between circ_0026344 and miR-645 was presented by Circinteractome online tool (Figure 4a). To confirm the combination between circ_0026344 and miR-645, miR-645 was successfully transfected into CRC cells (Figure 4b), and the noticeable decrease in the luciferase activity in circ_0026344 WT further exposed the existence of combination in CRC cells (Figure 4c and d). Meanwhile, miR-645 was upregulated in CRC cells compared with normal cells (Figure 4e). As expected, the deletion or overexpression of circ_0026344 led to a promotion or an inhibition in miR-645 expression in CRC cells (Figure 4f and g). Moreover, si-circ#1 could mitigate the curb which was triggered by propofol in miR-645 expression in CRC cells (Figure 4h and i). These data elucidated that circ_0026344 harbored miR-645 and negatively regulated miR-645 expression in CRC cells.

### 3.5 Propofol inhibited the proliferation and metastasis and promoted apoptosis by circ_0026344/miR-645 axis in CRC cells

**Figure 5:**
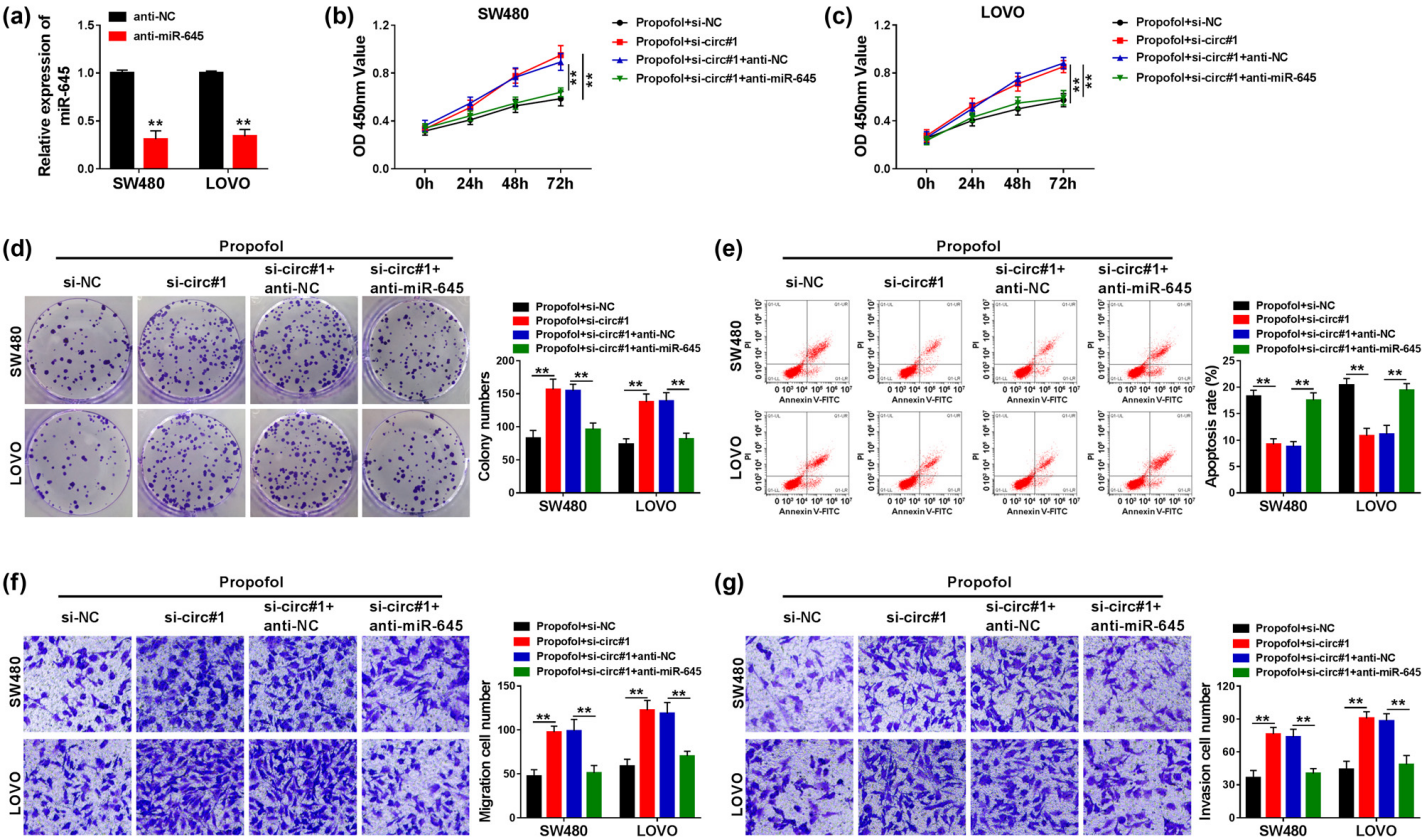
circ_0026344 regulated CRC development by binding to miR-645 in propofol-treated SW480 and LOVO cells. (a) miR-645 expression was detected by qPCR in SW480 and LOVO cells transfected with anti-NC or anti-miR-645. SW480 and LOVO cells were treated with propofol + si-NC, propofol + si-circ#1, propofol + si-circ#1 + anti-NC, and propofol + si-circ#1 + anti-miR-645, respectively. (b–d) Cell proliferation was determined by CCK8 and colony formation assays. (e) Cell apoptosis was detected by flow cytometry assay. (f and g) Cell migration and invasion were detected by transwell assay. Significant differences were compared with Student’s *t*-test (a) or ANOVA (b–g). **P < 0.01.

Based on the relationship between circ_0026344 and miR-645, the functional regulation of circ_0026344 to miR-645 was further explored. The significant decrease in miR-645 expression indicated a successful transfection efficiency of anti-miR-645 in CRC cells (Figure 5a). Furthermore, anti-miR-645 abrogated si-circ#1-mediated augmentation in proliferation and metastasis in propofol-treated CRC cells (Figure 5b–d, f and g). In addition, si-circ#1-induced counteraction in propofol-mediated promotion of apoptosis could be dispelled by anti-miR-645 in CRC cells (Figure 5e). These data highlighted that circ_0026344 affected the efficacy of propofol by regulating miR-645 in CRC cells.

### 3.6 Propofol participated in CRC progression by circ_0026344/miR-645/Akt/mTOR signal pathway

**Figure 6:**
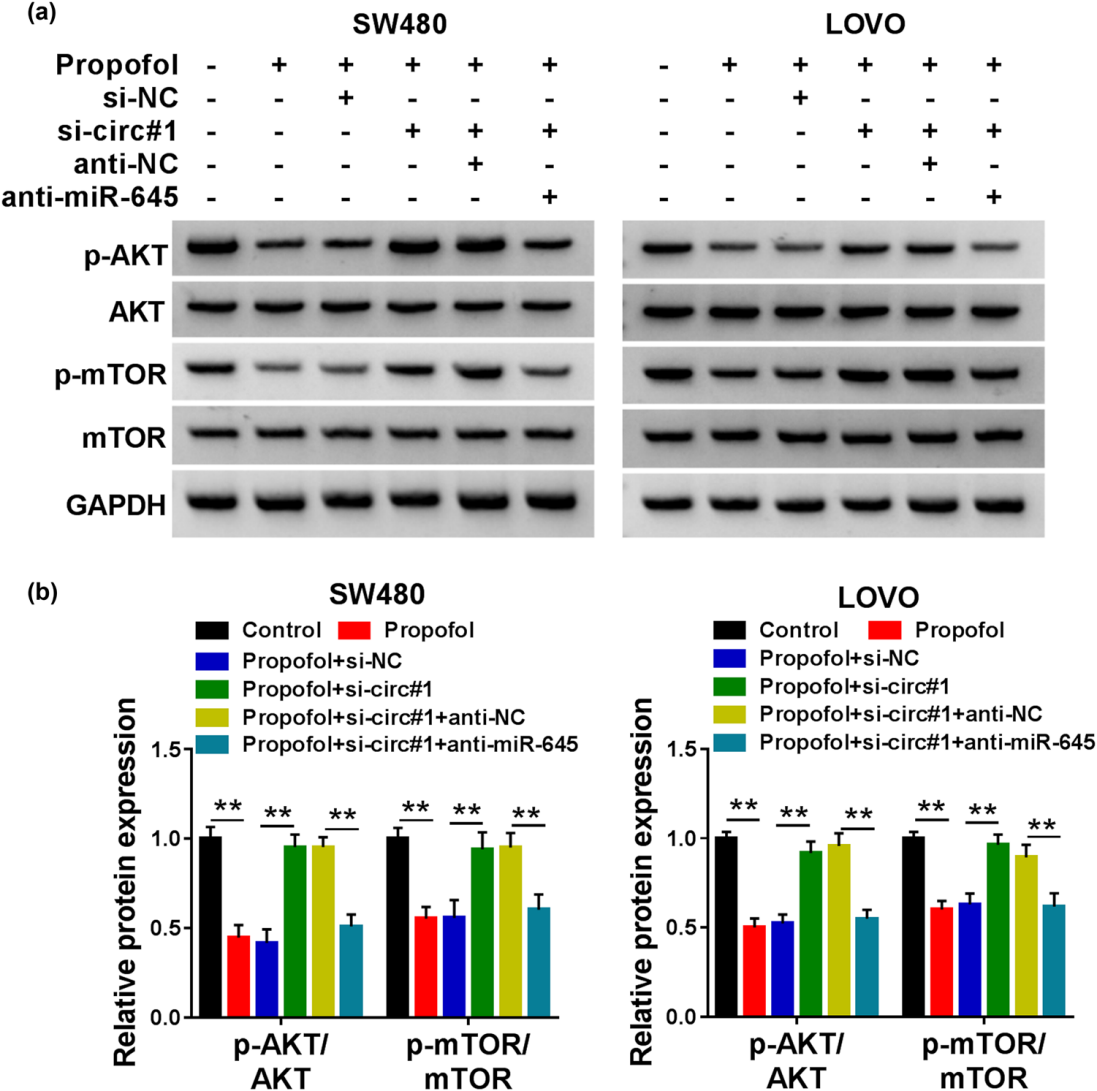
circ_0026344 silencing restrained propofol-inactivated Akt/mTOR pathway by interacting with miR-645. (a and b) The effects among propofol, circ_0026344 silencing, and miR-645 inhibitor on Akt/mTOR pathway were presented by detecting the protein levels of p-AKT, AKT, p-mTOR, and mTOR by western blot in SW480 and LOVO cells. Significant differences were compared with ANOVA. **P < 0.01.

The downstream signal pathway of circ_0026344/miR-645 was further explored in this study. Interestingly, the relative protein expression of p-AKT/AKT and p-mTOR/mTOR was strikingly impaired by propofol, which was ameliorated by si-circ#1; however, anti-miR-645 could abrogate the amelioration induced by circ_0026344 deletion in CRC cells (Figure 6a and b). These data disclosed that propofol treatment could repress Akt/mTOR signal pathway by circ_0026344/miR-645 axis in CRC cells.

## 4 Discussion

Propofol was recognized as an antitumor in the treatment of cancers. Propofol possessed the free radical scavenging ability, which was instantiated in the amelioration of ischemic brain injury by activating Akt/mTOR signal pathway [26]. In CRC, propofol interacted with NMDAR/CAMKII/ERK signal pathway to inhibit the glycolysis, thus hindered the development of CRC [27]. Meanwhile, propofol regulated miR-124-3p/AKT3 axis to restraint the proliferation in CRC cells [11]. The treatment of propofol in CRC cells led to elevate the apoptosis [28]. In this study, a dose-dependent abatement in proliferation and metastasis and a dose-dependent augment in apoptosis were presented in CRC cells treated by propofol. These findings were in accordance with the above reports.

circRNAs were frequently recognized as biomarkers of cancers. circ_0026344 was downregulated in CRC cells and has been suggested to be a biomarker of CRC on account of the correlation between circ_0026344 expression and the prognosis [29]. Moreover, circ_0026344 downregulation suppressed the migration and invasion of CRC cells [30]. Consistently, circ_0026344 was lowly expressed in CRC cells compared with normal cells; meanwhile, circ_0026344 overexpression inhibited the proliferation and metastasis and boosted apoptosis in CRC cells. Interestingly, si-circ_0026344 allayed propofol treatment-induced impediment in proliferation, metastasis, and augmentation in apoptosis in CRC cells. circRNA 001372 inhibition reversed the suppression in apoptosis, inflammation, and promotion of proliferation, which were induced by propofol in neuroinflammation [31]. In addition, circ_0026344 sponged miR-645 to regulate the proliferation, metastasis, and apoptosis. circ_0026344 sponged miR-21/miR-31 to restraint CRC development [29].

Increasing evidences exposed the role of Akt/mTOR signal pathway in regulating diseases’ development. Fan et al. illustrated that Akt/mTOR activation hampered the immoderate autophagy to ameliorate the myocardial damage [23]. Akt/mTOR inhibition restrained the proliferation of CRC [25]. In this study, propofol treatment-induced suppression in Akt/mTOR was reversed through si-circ_0026344; however, anti-miR-645 restored the suppression in CRC cells. FOXD2-AS1 harbored miR-195 to activate Akt/mTOR pathway to activate esophageal squamous cell carcinoma progression [32]. PRMT6/miR-372-3p/Akt/mTOR signal pathway facilitated endometrial cancer process [33]. These data clarified that circ_0026344 sponged miR-645 to restrain Akt/mTOR pathway in propofol-treated CRC cells.

The disadvantage of this paper is the lack of *in vivo* study that propofol can mediate tumorigenesis via circ_0026344 *in vivo*. And we will study that in subsequent study.

To sum up, circ_0026344 was downregulated and circ_0026344 overexpression or propofol treatment led to suppress the proliferation and metastasis and facilitate the apoptosis in CRC cells. In addition, propofol-induced amelioration in CRC development was assisted by circ_0026344/miR-645/Akt/mTOR signal pathway. Our findings not only provide a theoretical basis for further studying CRC therapy with propofol, but also present potential in treating CRC with propofol combined with circRNA-mediated drug.
